# Multiple Micronutrient Supplements will not Reduce Incidence of Low Birthweight

**DOI:** 10.4103/0970-0218.51221

**Published:** 2009-04

**Authors:** Umesh Kapil

**Affiliations:** Professor of Public Health Nutrition, All India Institute of Medical Sciences, New Delhi, India. Email: umeshkapil@yahoo.com

Low Birthweight (LBW) is multifactorial in etiology. Nearly 50 risk factors have been evaluated for their role in causing prematurity and intrauterine growth retardation. Statistically significant associations have been documented for several of them. Some of the critical Public Health Interventions suggested to reduce LBW include, delaying child bearing in adolescents, efforts to improve the nutritional status of women, particularly anemia in pregnancy, access to antenatal care, advice on adequate rest during pregnancy, especially in undernourished women, malaria prophylaxis or treatment in endemic areas, efforts to stop smoking and reduce tobacco chewing in areas wherever it is a common practice, improving female education, especially that of mothers, improvement in sanitation and water supplies, and so on.(1)

Presently, about 22% of the babies born in India are of low birthweight (less than 2.5 kg).(2) This figure has remained more or less stationary for the last few decades in spite of striking declines in neonatal and infant mortality. In the developed countries, a majority of LBW infants are preterms, whereas, in developing countries the reverse is the case. In India, a majority of newborns with LBW are full-term infants who are small for gestational age.(1) No matter how convincing the scientific data is, that a given factor is causally related to intrauterine growth or gestational duration, there is no evidence that its elimination or reduction will lead to a reduction in the incidence of LBW. The problem of LBW is multidimensional, and hence, no specific vertical program can be formulated to address this issue.

Micronutrient deficiencies among pregnant women are widespread in low-income countries, including India.(3,4) Among the underprivileged populations, pregnant women have limited access to food, and hence their dietary intake of calories, protein, vitamins, and micronutrients is inadequate. In recent times, selected international and bilateral groups have been advocating multiple micronutrient supplementations daily, through a capsule containing about 15-17 micronutrients, to all pregnant women, to reduce the incidence of LBW.(5) Several efficacy trials on multiple micronutrient supplementations to pregnant women in different parts of the world have shown conflicting results.(6–9) Adequate effectiveness data are also lacking on the impact of multiple micronutrient supplementations in improving birthweight and pregnancy outcomes.

In India, 11.4% of the women in the reproductive age group are below 145 cm, and 35.6% suffer from chronic energy deficiency (BMI less than 18.5). About 58.7% of pregnant mothers suffer from anemia.(2) It is not clear how a cocktail of multiple micronutrients can improve birthweight. The issues related to interactions between micronutrients and the coexistence of micro- and macronutrient deficiencies require serious consideration, before multiple micronutrient supplementations are given to pregnant women as a public health intervention in India.

There is growing evidence on the existence of metabolic interrelationships between different micronutrients, for example, magnesium, zinc, and calcium; copper and zinc compete with each other. The excess iron medication, in the absence of overall dietary improvement, can lower the zinc nutritional status. The addition of vitamin C improves the absorption of iron. The nutrient-nutrient interactions can be both positive and negative, and we do not have adequate scientific data on this aspect. Pregnant women of undernourished population groups have micronutrient deficiencies, which tend to coexist with macronutrient deficiencies. A pregnant woman will have coexisting deficiencies of zinc, iron, folate, and vitamin A along with a deficit in the intake of calories, protein, and fats. Under the multiple micronutrient supplementation strategy, supplementation of only vitamins and minerals is done and no effort is made to bridge the gap between the intake of calories and proteins. Also, biologically there is no plausible justification for the mechanism of action of vitamins and minerals alone in improving the birthweight of the newborn.

Diet of pregnant women in the poor income groups are deficient not only in micronutrients, but in energy as well.(10,11) What women require is food of good nutritive value and not just a capsule of selected synthetic nutrients. It has been appropriately questioned whether a single intervention is likely to cause a reduction in the incidence of LBW, which is largely dependent on the socioeconomic disparities accumulated over generations.(12) According to the World Health Organization (WHO) “there is no added benefit of multiple-micronutrient supplementation compared with supplementation with iron and folic acid alone. Iron and folate supplementation during pregnancy as recommended by the WHO should be implemented until more information is available”.(13)

Poor pregnancy outcome is the result of a multiplicity of factors and cannot be corrected by a narrow pharmaceutical shortcut. It calls for overall improvement in antenatal care and dietary diversification. This task cannot be evaded and there are no magic bullets. India is no barren desert. It is a country which can be rightly proud of its vast biodiversity. The challenge before Indian scientists is to investigate how best the vast array of foods that are available right at their own doorstep, and which are rich in several micronutrients, could be optimally used, in judicious combinations, to combat micronutrient deficiencies. A blunderbuss polypharmacy approach in the absence of scientific evidence will amount to exploitation of poor communities and will be putting an unnecessary strain on the already stretched resources of the health systems of our country.(14) Efforts should continue to adopt and improve the food-based approaches for sustainable solutions. The choice should be directed toward locally available and affordable solutions rather than those dependent on external sources.
